# Postpneumonectomy Syndrome: A Rare Case of Indolent Airway Collapse

**DOI:** 10.1016/j.jaccas.2024.102796

**Published:** 2024-12-04

**Authors:** Lindsey A. Braden, Daniel Quesada, Amber L. Jones

**Affiliations:** a Division of Acute Care and Trauma Surgery, Department of Surgery, Kern Medical Center, Bakersfield, California, USA; b Department of Surgery, Harbor-UCLA Medical Center, Torrance, California, USA; c Department of Emergency Medicine, Kern Medical Center, Bakersfield, California, USA

**Keywords:** cardiac computed tomography, dyspnea on exertion, mediastinal shift, pneumonectomy, postpneumonectomy syndrome, pulmonary circulation

## Abstract

Postpneumonectomy syndrome (PPS) is a rare postoperative phenomenon characterized by dynamic airway obstruction and circulatory collapse resulting from excessive mediastinal shifting and rotation of critical structures. This paper presents a novel case of PPS manifesting approximately 3 decades after pneumonectomy in an acutely symptomatic 28-year-old man with clinical findings concerning for impending airway collapse. Cardiac computed tomography and pulmonary function testing were used as alternative, noninvasive means of monitoring for disease advancement. Approximately 113 incidences of PPS appear in the literature. Although a diagnosis of exclusion, it is important to consider the phenomenon given the life-threatening consequences. For this reason, virtually no powered data investigating the rate of disease regression has been published in the medical literature to date. Although space-occupying surgery serves as the most frequently used treatment modality, there remains no consensus on PPS management, patient selection, or timing of intervention among thoracic societies.

## Graphical Abstract

**Figure:**
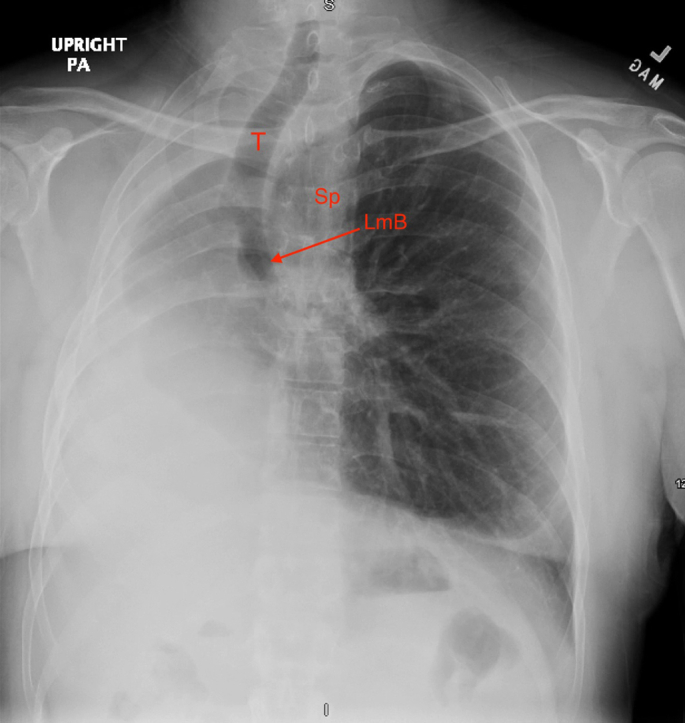

History of Presentation. A 28-year-old man presented with progressive shortness of breath for 20 days. The patient endorsed dyspnea with increasing functional limitations while completing normal daily responsibilities at his warehouse job. Additional complaints included orthopnea and dysphagia that often spontaneously resolved without interventions. On evaluation, he appeared in no distress, with unremarkable vital signs and no dyspnea at rest. Physical examination was concerning for dextrocardia including cardiac auscultation most prominent at the right chest and asymmetrical breath sounds that were diminished at the right hemithorax. No jugular venous distention, lower extremity edema, cyanosis, or digital clubbing was appreciated.

**Take-Home Messages**

• This paper illustrates a rare case of postpneumonectomy syndrome, a life-threatening but easily missed disease with a known insidious progression to complete airway and circulatory collapse.
• Future research efforts are needed to enhance understanding of the disease’s course to delineate optimal patient selection, timing, and surgical candidacy.

## Past Medical History

The patient’s medical history was significant for a right pneumonectomy at birth for a congenitally, nonviable right lung, and self-reported asthma. The patient underwent a right pneumonectomy as an infant. However, he had limited knowledge regarding his birth history and was unable to recall the indication for which pneumonectomy was completed. Additionally, the patient had not been medically evaluated since childhood and had received an arbitrary diagnosis of asthma from sporadic urgent care visits for intermittent episodes of shortness of breath.

## Differential Diagnosis

Differential diagnoses included pulmonary embolism, postsurgical reduced lung capacity, indolent infectious etiologies, and bronchopulmonary hyperactivity associated with asthmatic history including bronchiectasis and chronic obstructive pulmonary disease. Euvolemic heart failure, primary lung cancer, and secondary malignant pathologies with pulmonary metastases were also considered.

## Investigations

**Figure 1:**
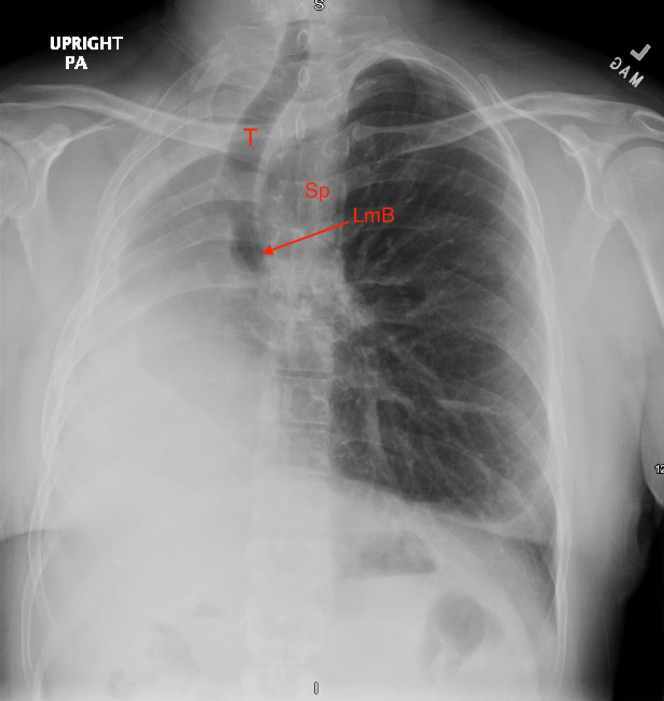
Chest Radiograph Depicting Complete Mediastinal Shifting in Postpneumonectomy Syndrome Anterior-posterior chest radiograph of a hyperinflated left lung with complete deviation of mediastinal contents including rightward shift of the trachea (T) and stretch of the left main bronchus (LmB, red arrow), past the vertebral column/spine (Sp).

**Figure 2:**
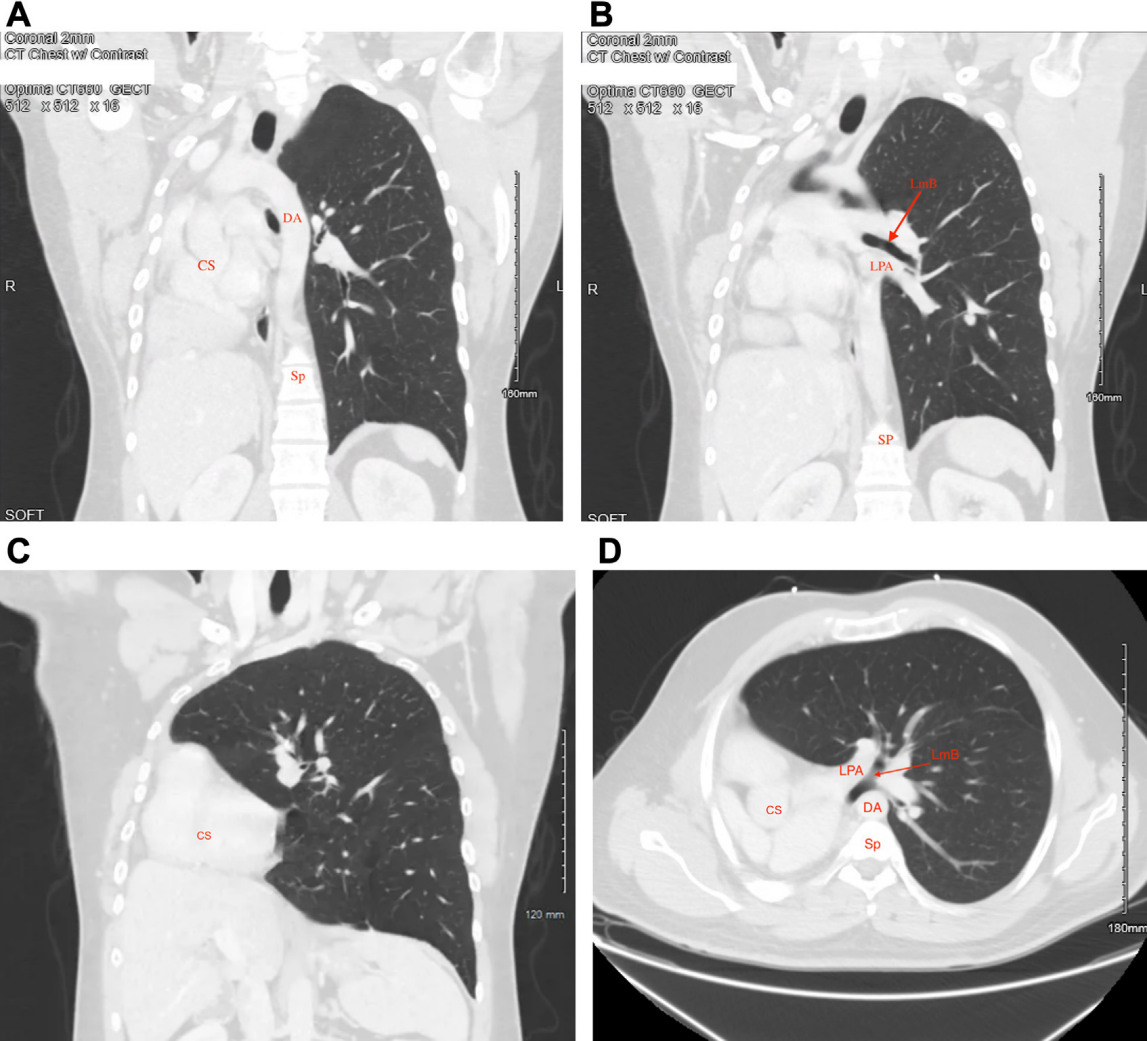
Computed Tomography With Intravenous Contrast of the Chest Showing Mediastinal Changes in Postpneumonectomy Syndrome (A-C) Coronal lung windows. (A) Herniation of the cardiac silhouette (CS) and segment 7 of the liver into the right hemithorax accompanied by supradiaphragmatic pleural thickening and a counterclockwise rotation of the aorta, at the level of the descending aortic arch (DA). (B) A patent left mainstem bronchus (LmB) and left pulmonary artery (LPA) shifted in a clockwise rotation around the aortic fulcrum. (C) Sequela of a remote right pneumonectomy with complete deviation of the mediastinum into the right hemithorax and hyperinflation of the left lung. (D) Axial view of the LmB traveling anterior to the thoracic spine (Sp) and posterior to the LPA.

**Figure 3:**
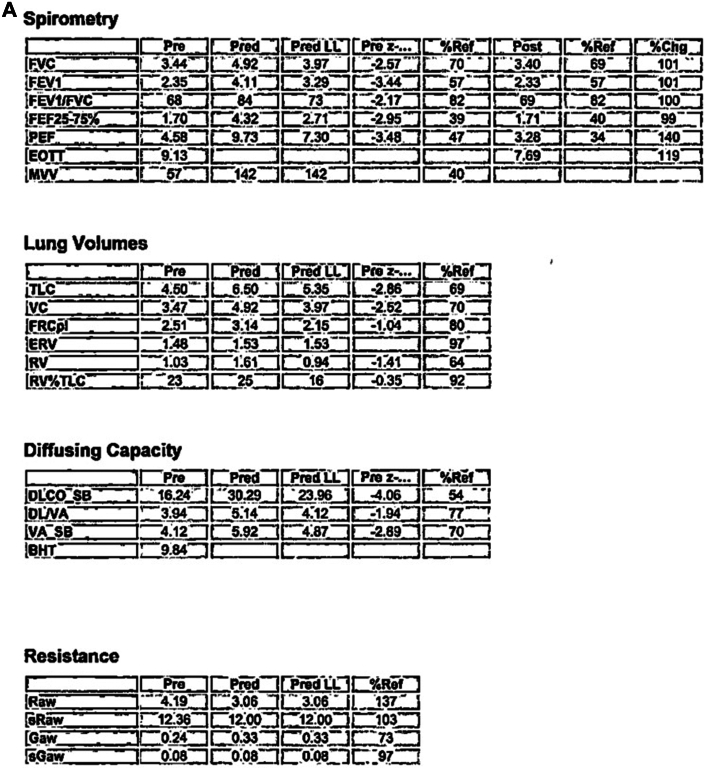

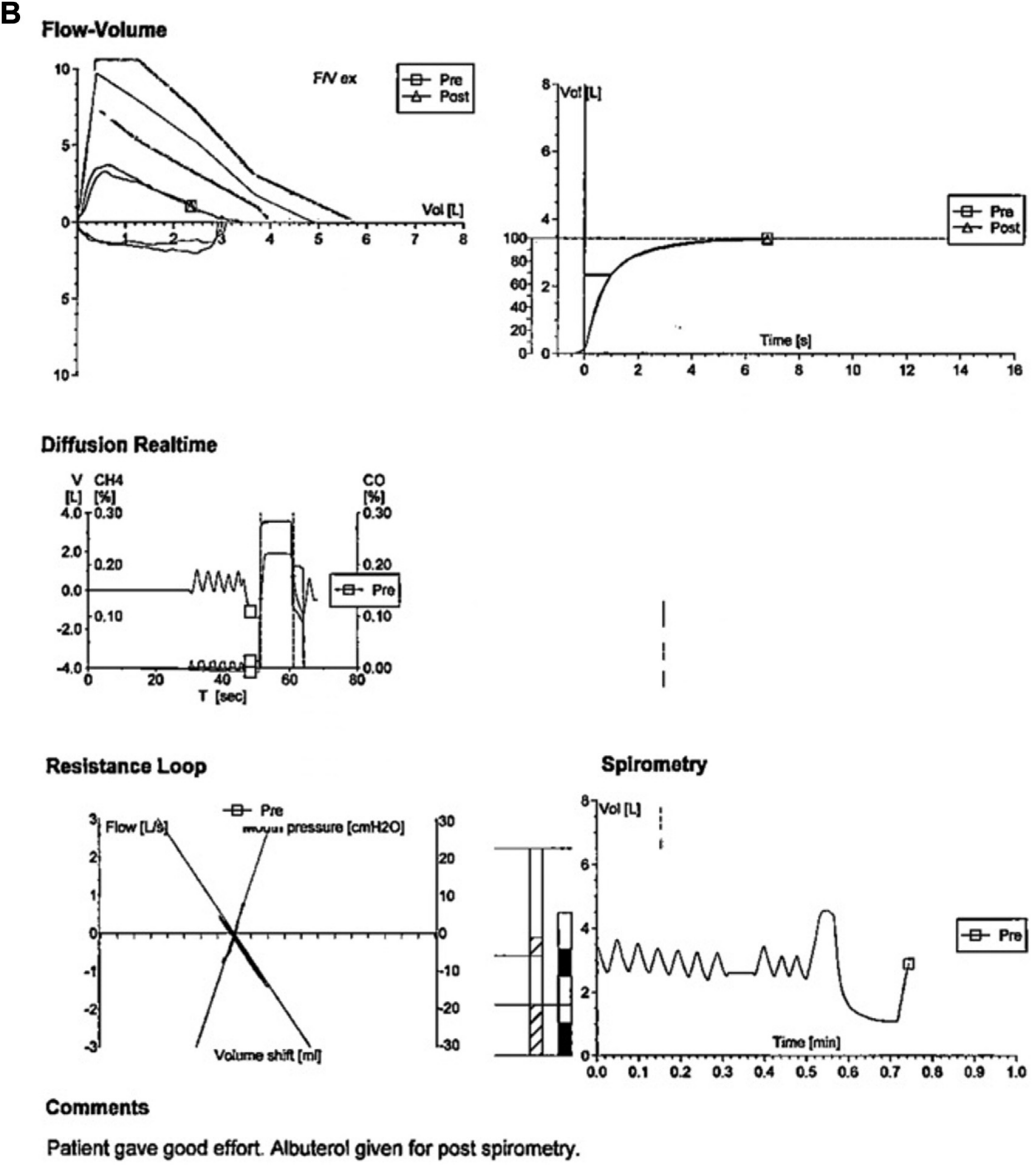
Pulmonary Function Testing Globally decreased pulmonary function values. No improvement in pre- to postbronchodilator (A) spirometry or (B) volume flow loop. BHT = breath holding test; DL/VA = diffusion capacity divided by alveolar volume; DLCO SB = diffusion lung capacity of carbon monoxide divided by alveolar volume in a single breath; EOTT = end of test; ERV = expiratory reserve volume; FEF = forced expiratory flow; FEV1 = forced expiratory volume in one second; FRCpl = functional residual capacity; FVC = forced vital capacity; Gaw = airway conductance; MVV = maximal voluntary ventilation; PEF = peak expiratory flow; Raw = airway resistance; RV = residual volume; RV%TLC = ratio of residual volume of total lung capacity; SB = accessible alveolar volume; sGaw = specific airway conductance; sRaw = specific airway resistance; TLC = total lung capacity; VC = vital capacity.

Initial chest radiograph revealed a hyperinflated left lung with mediastinal deviation into the right hemithorax, severe rightward tracheal deviation, and opacification of the right chest, most prominent over lung zone III (Figure 1). Chest computed tomography revealed sequela of remote right pneumonectomy, severe rightward tracheal and mediastinal deviation, and herniation of the hyperdistended left lung and the superior portion of the right hepatic lobe into the right hemithorax (Figures 2A to 2C). Also appreciated was a rightward counterclockwise rotation of the aorta and pulmonary artery with stretching of the left mainstem bronchus over the anterior aorta, passing posterior to the pulmonary artery (Figure 2D). Despite severe tracheal deviation, the tracheobronchial tree appeared patent with only slight narrowing. Pulmonary function testing (PFT) revealed increased resistance, ∼50% diffusion capacity, and globally decreased lung volumes (Figure 3A). Unsurprisingly, there was no interval improvement on postbronchodilator spirometry values (Figure 3B).

## Management

The patient was educated on the anticipated progression of disease, high probability of eventual airway collapse, and possible death. Investigation of the right hemithorax and eventual space-occupying surgical intervention for mediastinal repositioning was advised. He declined further intervention given his asymptomatic status at the time of evaluation and inability to abstain from work. However, the patient was agreeable to close outpatient follow-up and serial testing to monitor for disease progression.

## Outcome and Follow-Up

Immediate outpatient follow-up testing included transthoracic echocardiogram, 6-minute walk test, and serial PFT. Transthoracic transesophageal echocardiogram (TEE) revealed dextrocardia, mild systolic impairment, left ventricular ejection fraction of 45%, and no evidence of pulmonary hypertension. The 6-minute walk test revealed poor functional lung capacity and exercise tolerance as evidence by subjective and objective qualifiers, including moderate dyspnea on exertion (Modified Borg Scale score = 3; heart rate maximum, 103 beats/min at 5 minutes) and a low-to-very low predicted exercise capacity of 38.73% (expected total distance, 683.4 m; expected lower limit, 530.0 m; actual, 264.7 m). Given these findings, the risks and anticipated outcomes were reiterated. The patient ultimately decided to continue with close monitoring and continued to decline intervention.

Fifteen months after initial presentation, the patient remains with persistent dyspnea on exertion, described as waxing and waning nature, despite the addition of maintenance inhalers. He endorses symptomatic control with use of his rescue inhaler combined with rest. However, serial PFT continues to show no postbronchodilator improvement. Follow-up imaging remains unchanged, without any advancement of disease to date.

## Discussion

Postpneumonectomy syndrome (PPS) is a rare phenomenon characterized by dynamic airway obstruction resulting from extreme shifting of the mediastinum toward the empty hemithorax after pneumonectomy.^1^^,^^2^ Mediastinal displacement toward the pneumonectomy cavity results in great vessel rotation and lung hyperinflation and herniation. The tracheobronchial tree is ultimately compressed, leading to severely symptomatic central airway compression.^1^, ^2^, ^3^

The pathophysiology of PPS depends largely on laterality. After right pneumonectomy, PPS manifests as a clockwise mediastinal rotation toward the vacant hemithorax and counterclockwise rotation of the heart and great vessels, as seen in our patient. Mediastinal displacement is accompanied by a herniation of the hyperinflated lung, stretching the left mainstem bronchus across the thorax causing eventual compression at the descending aorta which acts as a fulcrum.^1^, ^2^, ^3^, ^4^ Few cases of PPS after left pneumonectomy are documented in the literature. Thus, theories regarding incidence and inciting pathology vary widely. However, the most favored theory involves a right-sided aortic arch serving as a precondition for disease development. Here a clockwise rotation of the heart and mainstem bronchus are stretched over the anterior vertebral column angulating bronchial vasculature.^2^, ^3^, ^4^ In both right and left PPS, intrathoracic displacement results in life-threatening compression of vital structures causing dynamic airway obstruction and hemodynamic instability due to decreased venous return from the pulmonary arteries and worsened by air trapping from the hyperinflated contralateral lung. Overall, current data on PPS outcomes have demonstrated a significant increase in morbidity and mortality if imaging manifestations remain untreated.^2^, ^3^, ^4^ However, the time frame in which these treacherous outcomes ensue remains unanswered.

Incidence of PPS ranges from 0.16% to 2%.^2^ For reasons that remain poorly understood, development can occur at any time interval after pneumonectomy and is seen in both children and adults.^4^ Incidence in adulthood remains unclear. However, development after pneumonectomy in childhood occurs in approximately 1 in 640 pneumonectomies.^1^, ^2^, ^3^, ^4^, ^5^ The childhood risk for indolent development is thought to be due to increased lung elasticity and compliance of the remaining tissues required for somatic growth.^2^^,^^4^

Symptoms of PPS are progressive in nature. In general, patients present with progressive dyspnea, cough, and inspiratory stridor, and often progress to respiratory compromise. As seen in our patient, esophageal compression remains a novel and a rare manifestation of disease. Tracheomalacia may develop due to prolonged compression, then thinning of the tracheal cartilage between the pulmonary artery and aorta or vertebral column.^1^

The timing and demographics associated with PPS remain unpredictable, complicating follow-up recommendations.^2^ Thus, given the indolent disease course, close monitoring at onset of potentially related symptoms with serial imaging is likely to be beneficial.^1^^,^^2^^,^^6^ There remains no consensus on optimal follow-up intervals after congenitally acquired pneumonectomy. Current recommendations are based on diagnostic reasoning for initial pneumonectomy. In a 30-year follow-up study completed by Laros et al,^6^ patients who underwent pneumonectomy from 0 to 5 years of age held a decreased risk for long-term postoperative complications when compared with patients >5 years of age. This is thought to be due to alveoli replication capability expected in this age group. Of note, symptomatic patients with pathognomonic changes on surveillance imaging underwent space-occupying intervention. No studies investigating timing between initial symptom development and complete airway collapse were found in the author’s literature search.

Diagnosis of PPS is made using imaging and PFT. As seen in our patient, posterolateral tracheal shift with mediastinal displacement toward the pneumonectomy site accompanied by a hyperinflated and herniated lung on chest radiograph significantly heightens concern for PPS (Figure 1). However, computed tomography remains essential for timely and accurate diagnosis, including detection of airway obstruction and the direction of heart and great vessel rotation.^1^, ^2^, ^3^^,^^6^ Lung function testing shows an increased resistance, decreased diffusion capacity, and lung volumes without postbronchodilator improvement.^1^, ^2^, ^3^ Work-up in our patient revealed severe tracheal shift and substantial reduction in exercise capacity. However, no active airway compromise was found, providing evidence for the progressive nature of PPS.

Data-driven treatment modalities addressing PPS remain sparse. Common interventions include tracheobronchial stenting and implantation of space-occupying prostheses.^3^ Other surgical techniques include aortopexy, tracheobronchial reconstruction, pericardiorrhaphy, pericardial flaps, sternal pericardial fixation, anterior vertebral body resection, or transposition of intercostal muscles.^4^^,^^5^ The most pursed intervention involves surgical repositioning of the mediastinum by filling of the postpneumonectomy space with nonabsorbable material (eg, saline breast prosthesis). In such cases, intrapleural prostheses are implanted, with the necessary volume determined intraoperatively, indicating that relief of bronchial compression remains central, over precise central positioning.^4^^,^^7^, ^8^, ^9^ Several studies suggest an inverse correlation between reduction in postoperative force expiratory volume in one second (FEV_1_) and degree of symptomatic improvement after this treatment.^1^^,^^2^^,^^10^ The therapeutic aspect is thought to be from restoring normal anatomic relationships, liberating the compressed airway to its patent position and ultimately returning the hyperexpanded lung to its correct location. Although some available literature shows statistically significant improvement, due to the sparsity of data, long-term outcomes of space-occupying therapy has not yet been established with certainty and necessitates future investigation.^1^, ^2^, ^3^, ^4^^,^^10^

Although our patient declined surgical evaluation, literature recounting similar cases note intraoperative challenges developing secondary to the chronicity of PPS. Such challenges involve development of dense scar tissue and require thoracotomy with lysis of adhesions prior to definitive fixation.^1^^,^^2^^,^^8^ Additionally, current research has focused on interventions once PPS is identified and lacks investigation on timing between initial symptom onset and complete airway collapse.

In cases of hemodynamic instability, TEE before or after correction may be of high value. TEE allows for visualization of cardiac strain, allows for confirmation of improved venous return, and may assess for decompensation from the implanted tissue expander.^8^^,^^10^ However, a high level of caution must be exercised given the risk of worsening mediastinal deviation, and esophageal compression or perforation from the sonography probe.^10^ In our patient, risks of TEE outweighed benefits, given that cardiopulmonary symptoms occurred with exertion only, coupled by his questionable esophageal compression.

## Conclusions

Although a rare and indolent diagnosis, PPS holds an exceptionally high risk of morbidity and mortality with catastrophic outcomes. The progressive nature of PPS may deter patients from pursuing intervention even when imaging changes manifest, possibly until airway collapse or lifesaving interventions are required. We propose that further efforts investigate the time frame between initial symptom onset and the progression toward airway collapse to better understand optimal patient selection and timing in which surgery should or should not be pursued. This remains an important unanswered question given that patients with certain socioeconomic constraints may be negatively affected unnecessarily by the medical field’s urgency for immediate correction, driven solely by the known catastrophic outcomes, but lack knowledge on when these may develop.

## Funding Support and Author Disclosures

The authors have reported that they have no relationships relevant to the contents of this paper to disclose.
